# Cervical squamous cell carcinoma of unknown primary: Oncological outcomes and prognostic factors

**DOI:** 10.3389/fonc.2022.1024414

**Published:** 2022-11-14

**Authors:** Jeroen Meulemans, Jens Voortmans, Sandra Nuyts, Jean-François Daisne, Paul Clement, Annouschka Laenen, Pierre Delaere, Charlotte Van Lierde, Vincent Vander Poorten

**Affiliations:** ^1^ Otorhinolaryngology, Head and Neck Surgery, University Hospitals Leuven, Leuven, Belgium; ^2^ Department of Oncology, Section Head and Neck Oncology, KU Leuven, Leuven, Belgium; ^3^ Radiation Oncology, University Hospitals Leuven, Leuven, Belgium; ^4^ Department of Oncology, Section Experimental Radiotherapy, KU Leuven, Leuven, Belgium; ^5^ Medical Oncology, University Hospitals Leuven, Leuven, Belgium; ^6^ Interuniversity Institute for Biostatistics and Statistical Bioinformatics, Leuven, Belgium

**Keywords:** Carcinoma of unknown primary, CUP, oncological outcomes, prognostic factors, squamous cell carcinoma

## Abstract

**Background/Objectives:**

Cervical squamous cell carcinoma of unknown primary (SCCUP) is a rare entity within head and neck cancer and both treatment regimens as well as identified potential predictors for oncological outcomes vary between published series. In this study, we evaluated oncological outcomes and identified potential prognostic factors for outcome.

**Patients and methods:**

This retrospective monocentric cohort study includes 82 SCCUP patients diagnosed and treated between January 2000 and June 2021. Overall survival (OS), disease-specific survival (DSS), disease-free survival (DFS) and locoregional recurrence-free survival (LRFS) were evaluated. The Cox proportional hazards model was used to analyze the prognostic effect of patient and tumor characteristics on oncological outcomes.

**Results:**

Five year OS, DSS, DFS and LRFS were respectively 53.9%, 72.2%, 68.9% and 67.3%. The p16 status was evaluated in 55 patients with 40% being p16 positive. On univariable analysis, p16 negative SCCUPs had significantly worse survival and recurrence rates in the presence of clinical extranodal extension (cENE) (OS: p=0.0013, DSS: p=0.0099, DFS: p=0.0164, LRFS: p=0.0099) and radiological extranodal extension (rENE) (OS: p=0.0034, DSS: p=0.0137, DFS: p=0.0167, LRFS: p=0.0100). In p16 positive SCCUP patients, rENE had a significantly negative prognostic effect on DFS (p=0.0345) and LRFS (p=0.0367). Total group multivariate analysis identified rENE as an independent negative predictor for all oncological outcomes. The “number of positive lymph nodes” was a second independent predictor for DSS (p=0.0257) and DFS (p=0.0435).

**Conclusions:**

We report favorable oncological outcomes, comparable to previously published results. Although the presence of rENE seems associated with poor oncological outcomes, the differential effect of clinical, radiological and pathological ENE in both p16 positive and negative subgroups remain to be elucidated by further prospective research.

## Introduction

Cervical squamous cell carcinoma of unknown primary (SCCUP) accounts for 2 to 5% of head and neck malignancies (1–3). SCCUP is a heterogeneous entity, defined by the presence of one or multiple metastatic cervical lymph nodes without evidence of a primary tumor in the upper aerodigestive tract (UADT), despite comprehensive diagnostic work-up (3–7). In the last two decades, the incidence of SCCUP has risen and parallels the increasing incidence of human papillomavirus (HPV) related oropharyngeal squamous cell carcinoma (OPSCC) (4, 5, 8). In oropharyngeal cancer, several series identified HPV positivity as a favorable prognosticator for survival when compared to HPV negative OPSCC (9, 10). Theories of SCCUP carcinogenesis include subclinical dormancy, undetectable location and spontaneous regression of the primary tumor site (11, 12). Standard diagnostic work-up of a potential SCCUP case consists of a comprehensive clinical head and neck evaluation, flexible endoscopy of the UADT, preferentially augmented with bio-endoscopy such as narrow-band imaging (NBI), which can increase in-office detection with 35%, state of the art imaging and fine needle aspiration cytology (FNAC) or core needle biopsy (CNB) of the cervical adenopathy (1, 13, 14). Contrast enhanced computed tomography (CECT), magnetic resonance imaging (MRI) and positron-emission tomography (PET/PET-CT) are used following a step-wise approach, with PET/PET-CT before any UADT biopsy (15). HPV testing is performed after FNAC or CNB and Epstein-Barr virus (EBV) testing should be considered in HPV-negative metastases (1). In case of a persistent occult primary tumor despite the aforementioned diagnostic work-up, the upper aerodigestive tract is evaluated under general anesthesia (examination under anesthesia – EUA) by means of panendoscopy with or without directed biopsies and uni- or bilateral palatine tonsillectomy (2). Despite this approach, the primary tumor remains undetected in approximately 40% of patients (7). Recently, transoral laser microsurgery (TLM) and especially transoral robotic surgery (TORS) emerged as new modalities aiding in detection of the primary tumor site, potentially influencing definite treatment of SCCUP. As in most HPV-related SCCUP patients the primary lesion is located in the cryptic lymphoepithelium of the palatine or lingual tonsils, complete tongue base mucosectomy (TBM) is nowadays advocated, resulting in a primary detection rate of 53-78% (16, 17). Another new development which is recommended in the work-up of SCCUP patients is “Next Generation Sequencing” (NGS), i.e. genetic profiling of (DNA) sequences on histopathological tissue of the metastatic lymph nodes. This can help in predicting the primary tumor (e.g. identification of gene profiles associated with ultraviolet light damage which are more consistent with skin cancer) and could potentially reveal the presence of any drug-actionable genomic alterations (18–20). Treatment of SCCUP is focused on controlling cervical nodal disease as well as eradicating the occult primary tumor. Locoregional control is attempted through single or bi- and triple-modality therapy, yet the optimal treatment regimen is still controversial because of conflicting previously published data (21–24). In this paper we report the results of a tertiary referral center (University Hospitals Leuven, Belgium) retrospective cohort analysis with assessment of oncological outcomes and identification of prognostic factors for outcome in SCCUP patients.

## Patients and methods

### Patients

This monocentric, retrospective cohort analysis was approved by the Institutional Review Board (University Hospitals Leuven Committee for Medical Ethics, study number MP017871). No informed consent was needed because of the retrospective setting. Multidisciplinary tumor board reports were queried for patients with potential SCCUP diagnosis (registered as cTx/T0 N+) between January 1, 2000 and June 17, 2021. Medical records of individual patients were screened in detail before in- or exclusion.

Predefined inclusion criteria were:

- Histopathological proven cervical SCC
- Failure of primary tumor detection after a comprehensive clinical examination, imaging (CT, MRI and/or PET-CT) and panendoscopy of the UADT including direct/indirect laryngoscopy +/- biopsies (cT0N+) and rigid/flexible oesofagoscopy.

Predefined exclusion criteria were as follows:

- Non-SCC
- Previous therapy for a head and neck malignancy
- Synchronous malignant primary tumor elsewhere (second primary)
- Treatment and/or substantial follow-up elsewhere than the University Hospitals Leuven

In our study population of SCCUP patients, 3 main groups were defined:

- cT0N+ SCCUP without identified primary tumor after standard work-up (cfr supra), consisting of clinical examination, panendoscopy and state of the art imaging
- cT0N+ SCCUP without identified primary tumor after standard work-up + therapeutic palatine tonsillectomy +/- TBM (pT0N+)
- cT0N+ SCCUP with eventual detection of the primary tumor after previous surgical interventions (palatine tonsillectomy +/- TBM)(pT+N+)

### Study endpoints

The primary objective of this study was the evaluation of oncological outcomes and identification of possible prognostic factors for outcome. Secondary endpoints were: description of patient, tumor and treatment characteristics; functional outcomes; primary detection rate and (de-)intensification of adjuvant therapy after TORS/TLM treatment of SCCUP patients including TBM.

### Data

After assessing for eligibility, the medical records of included patients were screened in a retrospective manner. All data were pseudonymised, with central storage in an online REDCap (Vanderbilt University, Nashville) database under control of the University Hospitals Leuven. We collected data regarding patient, tumor and diagnosis/treatment characteristics and functional and oncological outcomes. Patient characteristics contained information about age, gender, follow-up and smoking and drinking status. For diagnostic work-up, PET-CT and examination under anesthesia (EUA) were evaluated. Tumor characteristics included HPV status, clinical (cTNM) and pathological (pTNM) TNM-classification and presence/absence of extranodal extension (ENE). ENE was subdivided in: pathological ENE (pENE) which was extracted from the pathology report; radiological ENE (rENE) visible on CT or MRI and clinical ENE (cENE) characterized by involvement of the overlying skin and/or the deep neck structures (fixation upon clinical examination) as well as by presence of neuro-vascular impairment. HPV testing was performed using p16 immunohistochemistry, using p16 as a surrogate marker for HPV. Patients were staged following the Union for International Cancer Control (UICC) TNM 7^th^ edition (25). If p16 status was known, an additional UICC TNM-8 staging was included (26). The site of origin and section margin status were reported and evaluated if the primary tumor could be identified by TLM/TORS. Treatment characteristics comprised primary and adjuvant disease management. For primary surgery, we differentiated between: isolated neck dissection (ND); ND with conventional palatine tonsillectomy; ND with TBM, with or without palatine tonsillectomy, by means of TORS/TLM (1). We distinguished between diagnostic and therapeutic palatine tonsillectomy: the former is considered as a part of diagnostic work-up, before primary surgery and often performed in another center prior to referral of the patient to our tertiary referral center. The latter is performed together with a ND as a part of primary surgical therapy (2). We gathered information about the extent of ND, number of resected and metastatic lymph nodes and surgical complications. Postoperative hemorrhage was defined as the need for surgical revision under anesthesia; minor and major wound infection were local infections with need for administration of antibiotics or surgical drainage respectively. Functional outcomes covered the need for tracheotomy, gastrostomy-tube feeding and duration of both.

### Definition of oncological outcome measures and statistical analysis

Overall survival (OS) is the time between diagnosis and death of any cause. Patients alive are censored at last follow-up. Disease-specific survival (DSS) is the time between diagnosis and disease-related death. Patients alive are censored at last follow-up as well. Disease-free survival (DFS) is the time between diagnosis and the earliest among recurrence of any type (local, regional, locoregional or distant) or disease-related death. Patients alive and disease-free are censored at last follow-up. Locoregional recurrence-free survival (LRFS) is the time between diagnosis and the earliest among local and/or regional recurrence or disease-related death. Patients alive and local/regional relapse-free are censored at last follow-up. Non-disease-related death is considered as a competing event for DSS, DFS and LRFS.

Residual disease is defined as the persistence or relapse of disease within 6 months after primary therapy. Recurrence of disease is the relapse of local, regional, locoregional or metastatic disease after more than 6 months and no more than 5 years following treatment of the primary tumor. We defined local recurrence as the appearance of a tumor in the UADT (oral cavity, oropharynx, larynx, hypopharynx and nasopharynx). Regional recurrence is the appearance of a new lymphadenopathy (LA), ipsi- or contralateral of initial disease and locoregional recurrence is the appearance of a tumor in the UADT together with a LA. A secondary primary tumor in field is the occurrence of tumor in the UADT more than 5 years after diagnosis of the initial tumor.

Follow-up summary statistics were based on the Kaplan-Meier estimate of potential follow-up. The Kaplan-Meier method was used for estimating OS. The cumulative incidence function approach was used for DSS, DFS and LRFS accounting for non-disease-related death as competing event. The Cox proportional hazards model was used to analyze prognostic effects of patient and tumor characteristics on OS, DSS, DFS, LRFS. These results are presented as hazard ratios (HR) with 95% confidence intervals (CI). A forward selection procedure was used for multivariable analysis of independent prognostic variables on oncological outcomes, with a 5% level of significance for variable entering. All tests are two-sided, and a 5% significance level was assumed for all. No corrections for multiplicity were performed due to the explorative nature of the study. Analyses have been performed using SAS-software (version 9.4 of the SAS System for Windows).

## Results

### Patient characteristics

In total, 82 patients met the inclusion criteria and were included in the analysis. At time of diagnosis, mean age was 62.3 years. The majority of patients had a history of smoking (n=69) and alcohol use (n=68). More details are displayed in **Table 1**. During diagnostic work-up, 79 (96.3%) patients underwent PET-CT. EUA was performed in 42 (51.2%) patients because of insufficient in-office endoscopic visualization of the UADT. Diagnostic palatine tonsillectomy was performed in 5 (6.1%) patients, while 34 (41.4%) patients underwent a palatine tonsillectomy concurrent with the therapeutic neck dissection (defined as ‘therapeutic’ palatine tonsillectomy, cfr supra). Twenty-nine (35.4%) patients underwent a palatine tonsillectomy before SSCUP diagnosis and 14 patients (17.1%) did not get a tonsillectomy of any kind.

**Table 1:** Patient characteristics.

Patient characteristics	n	%
1. Gender (N=82)		
Male	62	75.61
Female	20	24.39
2. Age at time of diagnosis (N=82) (years)		
Mean	62.24	
SD	9.28	
Median	62.39	
IQR	(56.95; 67.40)	
Range	(35.83; 83.49)	
3. Smoking history (N=79)		
Never	10	12.66
Former	29	36.71
Current	40	50.63
4. Packyears smoked (N=68)		
Mean	37.79	
SD	23.17	
Range	(1.00 - 110.00)	
5. Ethyl use (N=81)		
Never	13	16.05
Past	21	25.93
Active	47	58.02
6. Ethyl units/day (N=66)		
<1, 1 or 2	19	28.79
3 or 4	12	18.18
5 or 6	12	18.18
7 to 9	7	10.61
10 or more	16	24.24

### Tumor characteristics

**Table 2** depicts tumor characteristics according to the 7^th^ and 8^th^ edition of the UICC-TNM staging system. All tumors were histologically confirmed SCC. P16 status was known in 67.1% (n=55) of patients of whom 40.0% (n=22) and 60.0% (n=33) were p16-positive and -negative, respectively. EBV status was not routinely determined. In total, 44 (53.7%) patients had ENE of any kind (cENE, rENE, pENE) with rENE in 30 (36.6%) and cENE in 21 (25.6%) patients. All patients with cENE had rENE as well. After ND, pENE proved present in 31 (48.4%) surgically treated individuals. Clinical staging was performed according to UICC-TNM 7^th^ edition (n=82), with most patients having advanced stage disease on initial presentation (79.3% cN2, 75.6% stage IVa). Four individuals had stage IVc disease, due to presentation with distant metastasis. Patients with known p16 status (n=55) were retrospectively restaged according to the UICC-TNM 8^th^ edition staging system, resulting in downstaging of p16-positive SCCUPs. Pathologically, 43.8% of these patients (n=21) were downstaged to stage I/II. Primary tumor detection was achieved in 5 (6.1%) out of all patients, with confirmed primary tumor location in the palatine tonsils (n=3) and base of tongue (n=2). All primaries were identified by TORS/TLM TBM with palatine tonsillectomy.

**Table 2:** Tumor characteristics.

CLINICAL STAGING*								
Variable	Total		p16 not tested (N=27)		p16 positive (N=22)		p16 negative (N=33)	
	n	%	n	%	n	%	n	%
1. Distant metastasis (N=82)								
M1	4	4.88	2	7.41	0	0.00	2	6.06
2. cN, 7th edition (N=82)								
N1	9	10.98	4	14.81	1	4.55	4	12.12
N2a	18	21.95	4	14.81	6	27.27	8	24.24
N2b	42	51.22	12	44.44	13	59.09	17	51.52
N2c	5	6.10	4	14.81	1	4.55	0	0.00
N3	8	9.76	3	11.11	1	4.55	4	12.12
3. cN, 8th edition (N=55)								
N1	23	41.82			19	86.36	4	12.12
N2	2	3.64			2	9.09	0	0.00
N2a	6	10.91			0	0.00	6	18.18
N2b	11	20.00			0	0.00	11	33.33
N2c	0	0.00			0	0.00	0	0.00
N3	1	1.82			1	4.55	0	0.00
N3a	1	1.82			0	0.00	1	3.03
N3b	11	20.00			0	0.00	11	33.33
4. TNM 7th, clinical staging (n=82)								
III	9	10.98	4	14.81	1	4.55	4	12.12
IVa	62	75.61	19	70.37	20	90.91	23	69.70
IVb	7	8.54	2	7.41	1	4.55	4	12.12
IVc	4	4.88	2	7.41	0	0.00	2	6.06
5. TNM 8th, clinical staging (N=55)								
I	19	34.55			19	86.36	0	0.00
II	2	3.64			2	9.09	0	0.00
III	5	9.09			1	4.55	4	12.12
IVa	16	29.09			0	0.00	16	48.48
IVb	11	20.00			0	0.00	11	33.33
IVc	2	3.64			0	0.00	2	6.06
PATHOLOGICAL STAGING **								
Variable	Total (N)		p16 not tested (N=16)		p16 positive (N=21)		p16 negative (N=27)	
	n	%	n	%	n	%	n	%
1. pN, 7th edition (N=64)								
N1	6	9.38	2	12.50	1	4.76	3	11.11
N2a	19	29.69	3	18.75	7	33.33	9	33.33
N2b	32	50.00	7	43.75	12	57.14	13	48.15
N2c	4	6.25	3	18.75	1	4.76	0	0.00
N3	3	4.69	1	6.25	0	0.00	2	7.41
2. pT, 7th edition (N=64)								
T0	59	92.19	16	100.00	17	80.95	26	96.30
T1	3	4.69	0	0.00	2	9.52	1	3.70
T2	2	3.13	0	0.00	2	9.52	0	0.00
3. pN, 8th edition (N=48)								
N1	22	45.83			19	90.48	3	11.11
N2	2	4.17			2	9.52	0	0.00
N2a	5	10.42			0	0.00	5	18.52
N2b	6	12.50			0	0.00	6	22.22
N2c	0	0.00			0	0.00	0	0.00
N3a	0	0.00			0	0.00	0	0.00
N3b	13	27.08			0	0.00	13	48.15
4. pT, 8th edition (N=48)								
T0	43	89.58			17	80.95	26	96.30
T1	3	6.25			2	9.52	1	3.70
T2	2	4.17			2	9.52	0	0.00
5. TNM 7th, pathological staging (N=64)								
III	6	9.38	2	12.50	1	4.76	3	11.11
IVa	54	84.38	13	81.25	20	95.24	21	77.78
IVb	3	4.69	1	6.25	0	0.00	2	7.41
IVc	1	1.56	0	0.00	0	0.00	1	3.70
6. TNM 8th, pathological staging (N=48)								
I	19	39.58			19	90.48	0	0.00
II	2	4.17			2	9.52	0	0.00
III	3	6.25			0	0.00	3	11.11
IVa	10	20.83			0	0.00	10	37.04
IVb	13	27.08			0	0.00	13	48.15
IVc	1	2.08			0	0.00	1	3.70

* Every patient was clinically staged according to UICC-TNM 7th edition (N=82). Patients with known p16-status were additionally clinically staged according to UICC-TNM 8th edition (N=55).

** Every patient receiving surgical treatment was pathologically staged according to UICC-TNM 7th edition (N=64). Patients with known p16-status were additionally pathologically staged according to UICC-TNM 8th edition (N=48).

### Treatment characteristics

Sixty-four patients (78.0%) underwent primary surgery. Ten patients (12.2%) were primarily treated with definite RT (n=4, 4.9%) or CRT (n=6, 7.3%). The remaining 8 patients received palliative RT (n=2, 2.4%), palliative CTx (n=3, 3.7%) or best supportive care (n=3, 3.7%). Reasons for palliative treatment setting were: presence of metastatic disease (n=3) or presence of medical comorbidities preventing treatment with curative intent (n=5). Primary surgical treatment characteristics are shown in **Table 3**. In 3 (4,6%) patients, bilateral neck dissection was performed because of bilateral nodal involvement. The mean lymph node yield was 27.3 nodes (SD 14.6, range 1.0-70.0), with a mean of 2.4 metastatic nodes (SD 2.2, range 1.0-15.0) in pN+ patients. Postoperative complications occurred in 21 patients (32.8%): unintentional and non-tumor related damage to n.XI (n=2), n.X (n=1), marginal branch of n.VII (n=1), lingual nerve (n=1) and n.XII (n=6). Additionally, 5 patients needed surgical revision due to early postoperative hemorrhage. Minor and major wound infections were reported in 3 and 1 patients, respectively. Other reported complications were: Horner syndrome (n=2), radial nerve palsy from peroperative malpositioning (n=1), intraoperative iatrogenic mandibular fracture (n=1) during TORS with need for open reduction and internal fixation, chylothorax (n=1) and 1 unplanned admission to the intensive care unit because of respiratory failure. Overall, mean length of hospitalization was 6.0 days (SD 3.2, range 1-21).

**Table 3:** Surgical treatment characteristics.

Surgical treatment characteristics	n	%
1. Type of primary surgery (n=64)		
ND	22	34.38
ND + palatine tonsillectomy	14	21.88
ND + TORS/TLM	28	43.75
2. TORS/TLM (n=28)		
TBM	8	28.57
Palatine tonsillectomy	1	3.57
TBM + palatine tonsillectomy	19	67.86
3. Type of neck dissection (n=64)		
RND	6	9.38
ERND	2	3.13
MRND	32	50.00
SND	24	37.50

ND, neck dissection; ERND, extended radical neck dissection; MRND, modified radical neck dissection; RND, radical neck dissection; SND, selective neck dissection; TBM, tongue base mucosectomy; TORS/TLM, transoral robotic surgery/transoral laser microsurgery.

Fifty-six (87.5%) surgically treated patients received adjuvant RT (n=38) or CRT (n=18). Eight (12.5%) patients did not receive any adjuvant treatment. One patient prematurely stopped adjuvant RT on own initiative. Mucosal irradiation in the primary (C)RT group was: panmucosal (n=5), targeted on the oropharynx (n=1), hypopharynx (n=1) and hypo- and oropharynx (n=1). Targeted oropharyngeal mucosal irradiation was administered in 12.5% and 32.7% of patients treated with primary and adjuvant (chemo-)radiotherapy, respectively. Noteworthy, 49 (76.6%) out of 64 surgically treated patients would have received chemoradiotherapy if primary therapy was non-surgical determined on age (<70 years), nodal stage according to TNM 7 (cN2, cN3) and the absence of contra-indications for chemotherapy. Adjuvant CRT was administered to only 18 (28.1%) individuals after primary surgery and so adding chemotherapy to the postoperative treatment was avoided in 31 patients by performing primary surgery (reduction of 63%) (27).

### Functional outcomes

No tracheotomies were necessary during the course of treatment. Long-term gastrostomy (PEG)-tube feeding proved necessary in 8 (9.8%) individuals; 1 after initial surgery, 3 during or after primary radiotherapy and 4 at time of adjuvant (chemo-)radiotherapy. Overall mean duration of PEG-tube feeding was 226.0 days (median 120, range 0.0-837.0, SD 272.7). Comparison between patients receiving primary and adjuvant (C)RT showed a mean feeding-tube dependency of 354.3 days (median 140.0, range 86.0-837.0, SD 418.9) and 186.3 days (median 142.0, range 70.0-291.0, SD 144.8), respectively.

### Oncological outcomes and prognostic factors for outcome

One patient was excluded from oncological outcome analysis because of recent diagnosis with follow-up less than 2 months at time of data collection. Mean and median length of follow-up for patients alive at the end of follow-up were 54.1 and 41.5 months (SD 44.2, Range 1.3-177.7 months) respectively. Mean and median Kaplan-Meier estimates of potential follow-up were 83.5 and 65.3 months (IQR 33.6-122.2), respectively.

**Figure 1:**
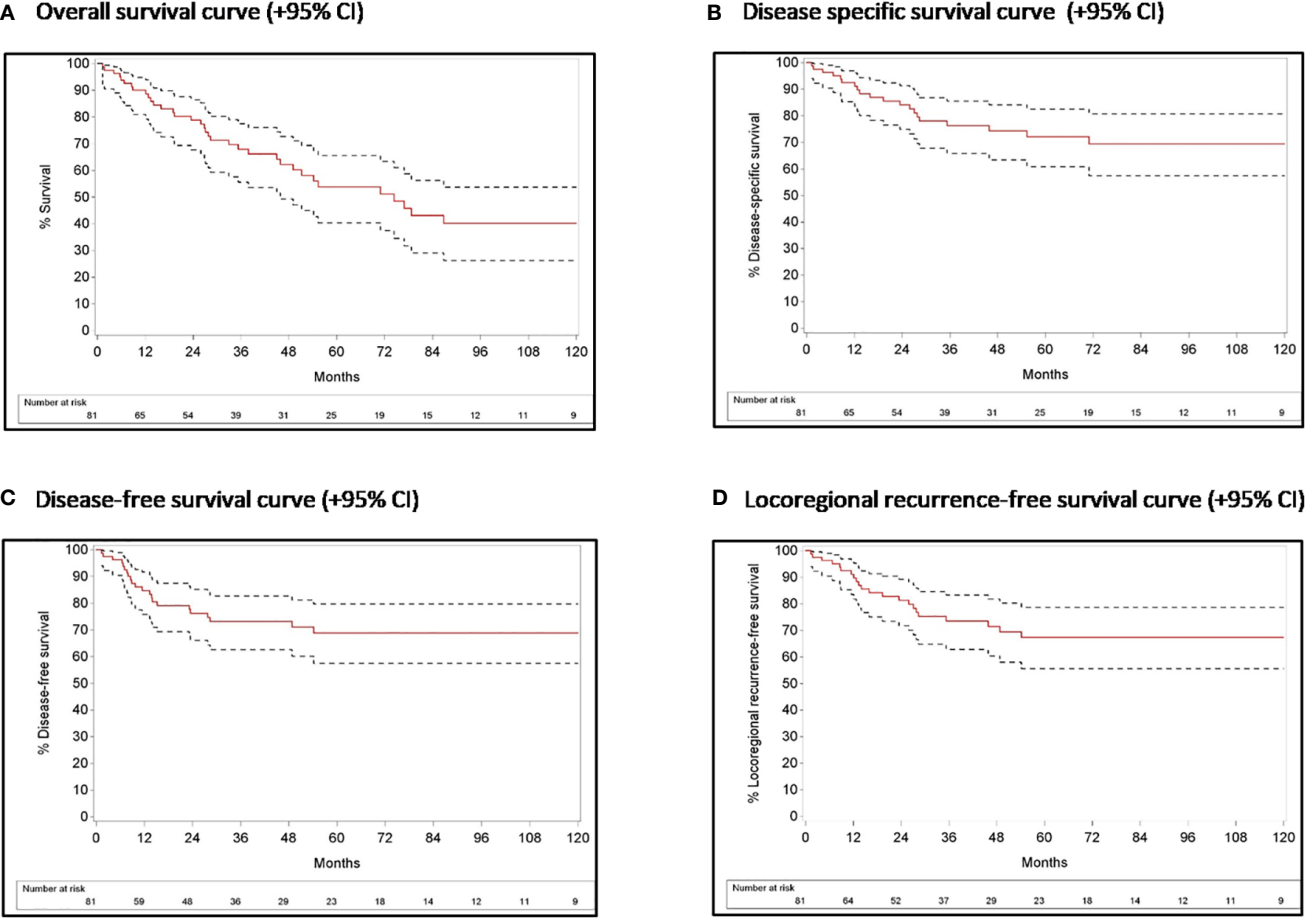
Survival curves with 95% confidence interval for the overall patient population.

**Figure 2:**
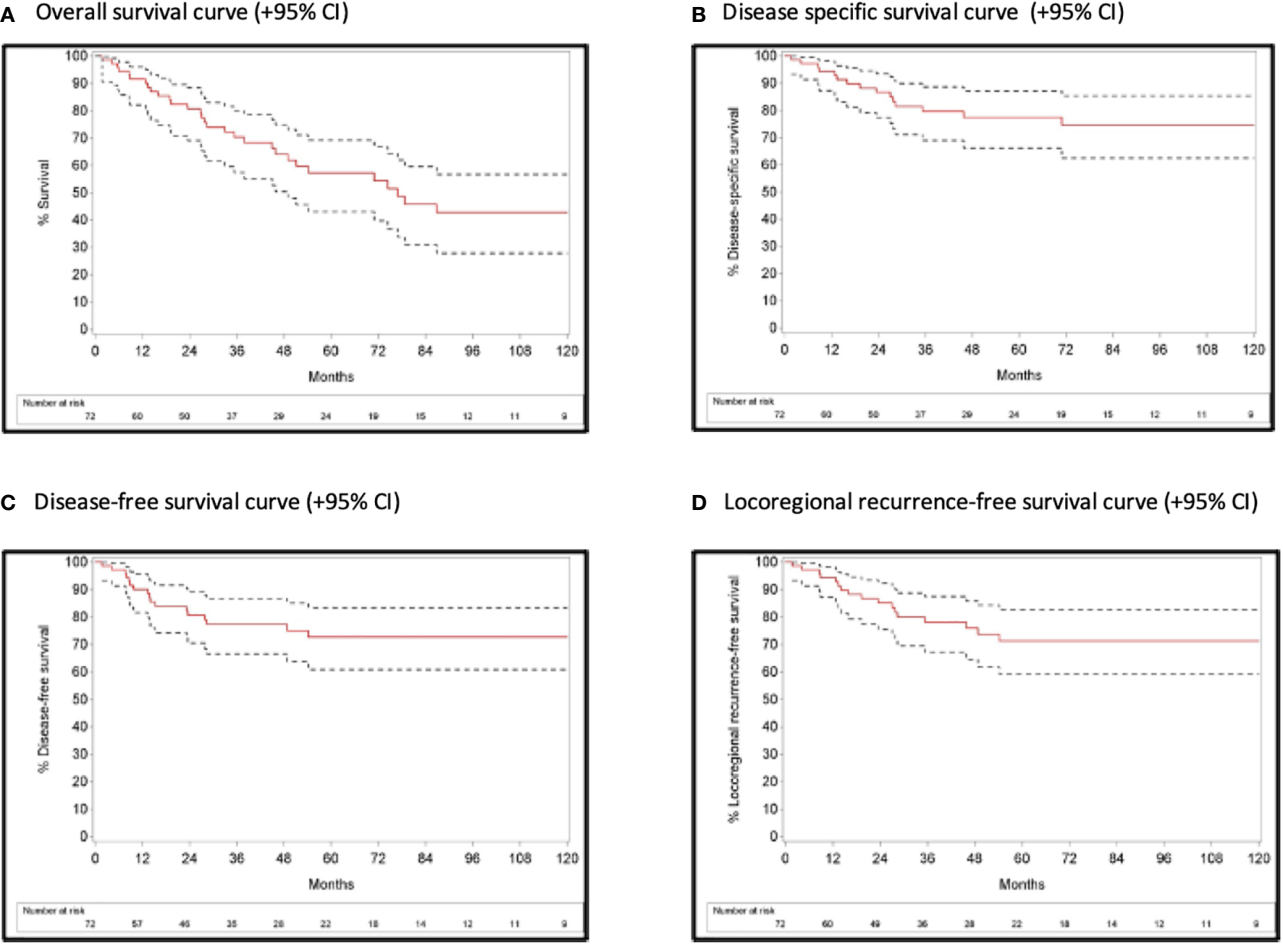
Survival curves with 95% confidence interval after exclusion of M+ SCCUP patients and SCCUP patients in whom the primary tumor was detected after palatine tonsillectomy +/- TBM (pT+N+).

In the total patient group (including patients with eventual detection of the primary tumor after palatine tonsillectomy +/- TBM as well as M+ patients), 22.2% of patients had residual disease and 12.4% developed disease recurrence (**Table 4**). A second primary tumor developed in 14 (17.3%) patients of which 4 were localized in the UADT. During follow-up, 40 (49.4%) patients died, 20 of them due to disease-related death. Seven patients died because of secondary primary disease. Two-year OS, DSS, DFS and LRFS [95% CI] were 78.2% [67.7%-86.5%], 84.1% [74.9%-91.3%], 76.3% [66.1%-85.2%] and 81.3% [71.7%- 89.3%], respectively. Five-year OS, DSS, DFS and LRFS were 53.9% [40.4%-65.6%], 72.2% [60.8%-82.5%], 68.9% [57.5%-79.7%] and 67.3% [55.6%-78.6%], respectively **(** **Figures 1A–D** **).** Details on treatment of residual and recurrent disease are depicted in **Table 4**. After exclusion of M+ SCCUP patients and SCCUP patients in whom the primary tumor was detected after palatine tonsillectomy +/- TBM (pT+N+) (n=73), two-year OS, DSS, DFS and LRFS [95% CI] were 80.8% [69.1%-88.4%], 86.7% [77.3%-93.5%], 80.7% [70.5%-89.2%] and 85.1% [75.5%- 92.4%], respectively. Five-year OS, DSS, DFS and LRFS were 57.3% [43.0%-69.2%], 77.5% [66.2%-87.1%], 72.7% [60.8%-83.4%] and 71.4% [59.1%-82.6%], respectively **(** **Figures 2A–D** **).**

**Table 4:** Disease relapse and treatment.

Disease relapse and treatment (N=81) *	n	%
1. Residual disease	18	22.22
Treatment (n=18)		
No treatment	10	55.56
eGFR-inhibitor (=cetuximab)	2	11.11
CTx	1	5.56
CTx + cetuximab (PFE)	2	11.11
Radiotherapy	1	5.56
Immunotherapy	2	11.11
2. Recurrent disease	10	12.35
Type of recurrence (n=81)		
No recurrence	71	87.65
Local tumor recurrence	1	1.23
Regional recurrence	2	2.47
Locoregional recurrence	1	1.23
Metastatic disease	6	7.41
Treatment (n=10)		
RT	2	20.00
Immunotherapy	1	10.00
CTx + cetuximab (PFE)	5	50.00
Surgery	2	20.00
Months after primary therapy (n=10)		
Mean	19.34	
SD	16.53	
Median	12.49	
IQR	(9.44; 20.23)	
Range	(6.28; 51.16)	
3. Second primary: In field	4	4.94
Location in UADT (n=4)		
Oral cavity	3	75.00
Oropharynx	1	25.00
4. Second primary: Out of field	10	12.35

CTx, Chemotherapy; EGFR, Epidermal growth factor receptor; RT, Radiotherapy.

PFE, Cisplatin + 5-Fluorouracil + Cetuximab; UADT, Upper Aerodigestive Tract.

*one patient was excluded for oncological outcome analysis due to limited follow-up.

Following variables were evaluated for their prognostic effect on oncological outcomes: p16 status, cN (TNM 7 and 8), pN (TNM 7), number of positive lymph nodes, and presence of cENE/rENE/pENE/ENE of any kind. The potential prognostic impact of p16 status was only assessed in the overall patient group, and the impact of cN status according to the 8^th^ ed TNM was only explored in the p16+ subgroup. **Table 5** depicts variables that proved significant prognosticators upon univariable analysis. Increasing cN status (TNM 7) was a negative prognostic factor for DFS and LRFS but not for OS and DSS in the total group. Presence of cENE and rENE were both negative prognosticators for OS, DSS, DFS and LRFS in the overall group as well as in the p16-negative subgroup. In p16-positive patients, however, this negative prognostic effect remained only significant for rENE in DFS (p=0.0345) and LRFS (p=0.0364). Concerning ENE of any kind (ENE-total), a negative prognostic effect was discovered for DSS (p=0.0359), DFS (p=0.0368) and LRFS (p=0.0364) but not for OS (HR 1.531 [0.789-2.969], p=0.2080) in total group analysis. ENE of any kind failed to reach significance in p16-positive and negative subgroup analysis. Surprisingly, p16-status had no significant prognostic effect on OS (HR 0.488 [0.189-1.259], p=0.1380), DSS (HR 0.420 [0.110-1.607], p=0.2052), DFS (HR 0.753 [0.245-2.319], p=0.6215) nor LRFS (HR 0.721 [0.234-2.225], p=0.5694). cN status (TNM 8) did not have a significant impact on oncological outcomes in the p16 positive subgroup. pN status (TNM 7) and presence of pENE did not have a significant impact on oncological outcomes, neither in the overall group nor in the p16 positive and negative subgroups. The number of pathologically confirmed metastatic lymph nodes turned out a borderline significant negative predictor for DFS (HR 1.176 [0.989-1.399], p=0.0672) in the overall group and for OS (HR 1.179 [0.993-1.399], p=0.0596) and DSS (HR 1.222 [0.985-1.516], p=0.0685) in p16 positive patients, with higher number of positive lymph nodes associated with poorer oncological outcomes. Upon multivariable analysis for the overall group, presence of rENE was confirmed an independent negative predictor for OS, DSS, DFS and LRFS. Number of positive lymph nodes was a second independent predictor for DSS (HR 1.234, p=0.0257) and DFS (HR 1.212, p=0.0435). After adding the latter to the multivariable forward selection model, rENE turned into a borderline significant prognosticator and non-significant prognosticator for DSS (p=0.0501) and DFS (p=0.1181), respectively.

**Table 5:** Overview of significant prognosticators upon univariable analysis for various oncological outcomes.

Variable	All patients	p16+ patients	p16- patients
cN, 7th edition #	DFS: HR 1.477 (p = 0.0386)	–	–
	LRFS: HR 1.494 (p = 0.0341)		
cENE *	OS: HR 2.148 (p = 0.0207)	–	OS: HR 9.873 (p = 0.0013)
	DSS: HR 2.845 (p = 0.0204)		DSS: HR 9.010 (p = 0.0099)
	DFS: HR 2.947 (p = 0.0120)		DFS: HR 7.509 (p = 0.0164)
	LRFS: HR 3.151 (p = 0.0077)		LRFS: HR 9.010 (p = 0.0099)
rENE *	OS: HR 2.440 (p = 0.0059)	DFS: HR 7.137 (p = 0.0345)	OS: HR 6.063 (p = 0.0034)
	DSS: HR 4.214 (p = 0.0023)	LRFS: HR 6.852 (p = 0.0367)	DSS: HR 7.678 (p = 0.0137)
	DFS: HR 3.993 (p = 0.0019)		DFS: HR 7.180 (p = 0.0167)
	LRFS: HR 4.264 (p = 0.0011)		LRFS: HR 8.407 (p = 0.0100)
ENE total *	DSS: HR 2.963 (p = 0.0359)	–	–
	DFS: HR 2.722 (p = 0.0368)		
	LRFS: HR 2.727 (p = 0.0364)		

HR, Hazard ratio; cN, clinical nodal classicifaction; cENE, clinical extranodal extension; rENE, radiological extranodal extension; ENE total, sum of cENE and rENE and pENE (pathological extranodal extension).

*These variables were analyzed as binary variables (Yes vs No). HR > (<) 1 means higher (lower) risk for ‘YES’ compared to ‘NO’ category.

#These variables were analyzed as ordinal variables: increased (decreased) risk with increasing predictor level (cN, pN: +1 level, number of LN: +1 node).

### TORS/TLM subgroup analysis

Twenty-eight CUP patients underwent TORS/TLM resection of the lingual/palatinal tonsils. The primary tumor (PT) was detected in 17.9% of these patients. There was no significant difference in tumor detection rates between p16-positive and p16-negative patients (p=0.133). Details are displayed in **Table 6A**. Section margins status of the primary tumor proved positive in 60.0% of patients. A comparison of adjuvant treatment characteristics according to PT detection is tabulated in **Table 6B**. PT identification by TORS/TLM did not significantly reduce the need for adjuvant radio(-chemo)therapy (p=0.824). Of the 5 patients with PT detection after TORS/TLM, 3 received adjuvant CRT because of cENE with negative resection margins of the PT (n=1), positive margins of the PT (n=1) and positive margins with additional pENE (n=1). One patient with contra-indications for chemotherapy received adjuvant RT for positive resection margins. In 1 patient, no adjuvant therapy was administered because of free-margins and stage I disease. However, PT identification did cause a significant reduction in the extent of mucosal radiotherapy (p=0.026): all 4 patients with detected PT who needed adjuvant RT/CRT, received local irradiation limited to the oropharynx and as such, panmucosal radiotherapy could be avoided. Bilateral irradiation of the mucosal and neck regions at risk was administered in 75.0% and 100.0% of patients after detection and non-detection, respectively.

**Table 6A:** TORS/TLM subgroup analysis: details on primary tumor detection according to p16 status.

Variable	Total (N)		p16-positive		p16-negative	
	n	%	n	%	n	%
Detection primary tumor	N=28		n=12		n=16	
No	23	82.14	8	66.67	15	93.75
Yes	5	17.86	4	33.33	1	6.25
Site origin	N=28		n=12		n=16	
BOT	2	7.14	2	16.67	0	
Palatine tonsil	3	10.71	2	16.67	1	6.25
Not found	23	82.14	8	66.67	15	93.75
Section margins	N=5		n=4		n=1	
Free (>2 mm)	2	40.00	2	50.00	0	
Close (0-2 mm)	0		0		0	
Positive	3	60.00	2	50.00	1	100.0

BOT, base of tongue, TLM, transoral laser microsurgery, TORS, Transoral robotic surgery.

**Table 6B:** TORS/TLM subgroup analysis: Comparison of adjuvant treatment characteristics according to primary tumor detection.

Variable	Primary not detected (n=23)		Primary detected (n=5)	
	n	%	n	%
1. Adjuvant treatment (N=28)				
No	6	26.09	1	20.00
RT	9	39.13	1	20.00
CRT	8	34.78	3	60.00
2. Side *				
Unilateral	0		1	25.00
Bilateral	16	100.00	3	75.00
3. RT on mucosal regions at risk *				
N	16	100.00	4	100.00
Extent				
Oropharynx	5	31.25	4	100.00
Panmucosal	11	68.75	0	0.00

RT. radiotherapy; CRT, chemoradiotherapy; TLM, transoral laser microsurgery; TORS. transoral robotic surgery.

*For the indicated variables, in the “primary not detected” group, one patient was excluded from analysis due to premature stop of radiotherapeutic treatment on own initiative (n = 16).

## Discussion

The primary objective of this retrospective study was to evaluate oncological outcomes and identify prognostic factors for outcome in a cohort of SCCUP patients. We observed a favorable 2- and 5-year OS of 78.8% and 53.9%, respectively. During follow-up, 18 patients (22.2%) had persistent disease after treatment and 10 patients (12.4%) suffered from disease recurrence. Eight patients developed distant metastasis, of whom 2 within 6 months. We observed a 2- and 5-year DFS of 76.3% and 68.9%, a 2- and 5-year DSS of 84.1% and 72.2% and 2- and 5-year LRFS of 81.3% and 67.3% respectively. Our reported 2-year OS corresponds to the 76-86% 2-year OS rate reported in previous studies (28, 29). On the other hand, our 5-year OS is in the lower range of the 5-year OS rates reported in several series during the last decade, with 5-year OS rates ranging between 54% and 85% (11, 28, 30–32). Our 5-year DFS (68.9%) is comparable with the 65-70% reported in recent published series (28, 30, 31). However, comparing these oncological outcomes needs caution. First, the exact definition of SCCUP varies among studies, leading to heterogeneous populations. Some authors defined a “clinical SCCUP” more liberally and included every patient with neck metastases and an occult primary site solely after in-office fiberoptic examination, without taking into account results of (nuclear) imaging (11). Others followed a similar comprehensive diagnostic work-up as we did, but only included patients treated with curative intent, which influences oncological outcomes as well (30, 32). A second reason why oncological outcomes between published series are difficult to compare is the variable rate of included HPV driven SCCUP patients. Ren et al. reported a significant difference in prevalence of HPV driven SCCUP in North America and Europe: in their meta-analysis (n=1149), they reported a better OS (HR 3.25 [2.45-4.31]) and DFS (HR 4.49 [2.88-7.02]) in HPV-positive compared to HPV-negative SCCUP patients (33). As such, one can assume that variability in the proportion of HPV-positive SCCUP patients within a study population may lead to inherent differences in oncological outcomes between populations. Taking the previous into account, Straetmans et al. defined their SCCUP cohort most similar to ours, however with a lower number of HPV-positive patients (7.8%). They reported a five-year OS of 54.9%, which is comparable to the 53.9% we reported (31).

Apart from analyzing oncological outcomes, this study aimed to asses potential prognostic effects on outcome of different variables. Upon univariable analysis of the total group, we identified a significant negative prognostic effect of increasing cN status (according to TNM 7^th^ ed) and of presence of ENE, including “ENE of any kind” (“ENE-total”: cENE and/or rENE and/or pENE), cENE and rENE. “ENE-total” proved a significant negative prognosticator for DSS, DFS and LRFS. Clinical ENE and rENE were negative prognosticators for all oncological outcome parameters in the overall patient group. Increasing cN status significantly reduced DFS and LRFS, but not OS and DSS. On multivariable analysis, an independent prognostic value of rENE for OS, DSS, DFS and LRFS could be confirmed. Additionally, the number of pathologically positive lymph nodes identified in the neck dissection specimen of surgically treated patients proved a second independent prognosticator for DSS (p=0.0257) and DFS (p=0.0435), as reported in previous series (34). Upon univariable analysis of population subgroups, cENE and rENE maintained their negative prognostic value in p16-negative patients. In p16-positive patients however, only rENE proved a negative predictor for DFS and LRFS. Although previously reported in other studies, p16 status, cN status (according to TNM 8^th^ ed), pN status (TNM 7^th^ ed) and pENE did not have a significant impact on different oncological outcomes, neither in the total group nor in the p16 positive and negative subgroups (9, 23, 28, 35). Whether p16-positivity is an independent positive prognostic factor in SCCUP, is still a matter of debate. It is generally accepted that HPV mediated/P16 + oropharyngeal SCC (OPSCC) has a significantly better prognosis than HPV-/p16- OPSCC, which translated into the recent UICC TNM 8^th^ edition staging system with TNM classification depending on HPV status (36, 37). However, also for SCCUP, several series reported p16-positivity as a significantly favorable predictor for OS (9, 32, 38–40) and DFS (10, 39). In a European multicenter study, Schroeder et al. reported an increase in HPV prevalence (1998-2014, p=0.007), with significantly improved OS and DFS in HPV driven SCCUPs (39). Also Dixon et al. reported improved DFS in HPV driven SCCUPs (10). Despite our hypothesis of its favorable prognostic value, p16-positivity could not be confirmed as a significant prognostic factor for outcomes in our cohort (OS (p=0.1380), DFS (p=0.6215), DSS (p=0.2052), LRFS (p=0.5694)). Other series were unable to prove an association between p16-positivity and more favorable oncological outcomes as well (28, 29, 34). However, our small number of patients with known p16 status might have been insufficient for powerful statistical analysis, as was previously stated in the series of Cho et al. (34) We conclude that there is a need for prospective, multi-institutional and international data to empower the debated prognostic significance of p16 status in SCCUP patients. This is of major importance, as adjuvant radiation dose de-escalation in the setting of HPV related OPSCC is currently under investigation (41).

Numerous preceding studies have suggested an association between presence of ENE and worse oncological outcomes in head and neck SCC (HNSCC) (42–44). However, conflicting results about the prognostic value of ENE in HPV-positive OPSCC have been published, with recent evidence demonstrating that pENE as well as rENE are associated with decreased OS and DFS in HPV-related and non-HPV-related OPSCC (43–48). This subdivision of ENE in pENE and rENE is clinically relevant, with rENE being a stronger negative prognostic factor for oncological outcomes than pENE (46). Concerning SCCUP, several studies reported a similar negative prognostic value of ENE for OS (9, 34), DSS (45), DFS (34) and LRFS (35, 45). However, most of these studies lack stratification by HPV status. Moreover, studies apply different definitions for ENE without clear differentiation between pENE, rENE and cENE. Our results demonstrate that presence of rENE had a significant negative prognostic effect in both HPV-related (DFS: p=0.0345; LRFS: p=0.0367) and unrelated (OS: p=0.0013; DSS: p=0.0099; DFS: p=0.0164; LRFS: p=0.0099) SCCUP patients. However, presence of pENE could not be identified as a negative prognostic factor, nor in the total SCCUP population, neither in the different subgroups. Currently, the 8^th^ edition UICC-TNM classification system adopts cENE and pENE as N-category modifiers for non-viral related HNSCC (37). However, Huang et al. stated that there are “compelling data demonstrating rENE as a powerful risk stratification tool to identify patients at high risk for treatment failure, especially distant metastasis, both in HPV-related and non-related HNSCC”, making it a possible parameter to refine cN classification in the future (48, 49). Our results can endorse the prognostic importance of rENE in HPV-related and unrelated HNSCC. Hitherto our study is the first to demonstrate the prognostic value of rENE in SCCUP patients. Despite this, there is need for standardization of taxonomy and universally accepted assessment criteria for rENE.

Twenty-eight SCCUP patients underwent TORS or TLM with lingual and/or palatine tonsillectomy. The primary tumor could be detected in 5 patients (17.9%) of whom 3 (60%) in the palatine and 2 (40%) in the lingual tonsils. This is remarkably lower than the PT identification rate of 32%, 53%, 74%, 74% and 80% described by Meulemans et al., Winter et al., Geltzeiler et al., Patel et al. and Hatten et al., respectively (5, 27, 50, 51). Although these series comprise a wide variety in preoperative diagnostic work-up and as such definition of SCCUP, a recent meta-analysis by Farooq et al. reported an identification rate of the PT of 73% with TORS/TLM after negative PET-CT (17). Another SCCUP-TORS meta-analysis (n=349) reported an average tumor identification rate of 70.8% (range 53-90), with 74.6% of primary tumors detected in HPV related SCCUP (52). In our group, 80.0% (n=4) of detected primaries were HPV driven: the considerable larger incidence of primary tumor detection in HPV related SCCUP might suggest a more important role of TORS in p16 positive patients, although sound evidence to support this finding is missing.

There are no randomized trials assessing the optimal adjuvant treatment after primary surgery (neck dissection with TORS/TLM) of SCCUP (1). In general, if the PT is detected by means of TORS/TLM resection of the palatine/lingual tonsils, the patient is strictly no longer considered as a SCCUP and should be treated accordingly, with margin status as one of the factors guiding adjuvant treatment. In our patient subgroup who underwent TLM/TORS, PT identification did not result in a significant reduction in the rate of adjuvant therapy (p=0.824). Moreover, trimodal therapy (surgery and adjuvant radiochemotherapy) was administered to 3 out of 5 patients with detected PT: adjuvant CRT was administered due to positive margins and/or ENE, according to current SCCUP ASCO guidelines (1). However, all patients with detected PT received isolated oropharyngeal irradiation which correlated with a significant RT volume reduction (p=0.026) when compared to patients without detected PT who often underwent panmucosal irradiation. Patel et al. suggested that surgical management of SCCUP with TORS can lead to deintensification of adjuvant therapy (avoidance of CTx, reduction of RT volume and dose) without increasing short term treatment failure (50). Current studies are examining the possibility of treatment de-escalation in HPV driven OPSCC. The ongoing PATHOS trial comprises a non-inferiority analysis of RT versus CRT in patients with a high-risk pathology profile (positive margins) after transoral surgery, as well as radiotherapy deintensification in patients with an intermediate-risk pathology profile (41). Future findings regarding deintensification might be extrapolated to HPV-positive SCCUP patients with eventual detection of the primary tumor in the oropharynx,. However, awaiting robust prospective results, adjuvant management should remain unchanged.

Whether the optimal treatment of SCCUP is primary surgery with adjuvant (chemo)radiotherapy or definite (chemo)radiotherapy is still a matter of debate (8). Wallace et al. reported improved regional control with ND on multivariable analysis (21). Amsbaugh et al. found better local (p=0.003) and loco-regional (p=0.068) recurrence free survival in patients treated with primary ND followed by RT/CRT (24). Demiroz et al. reported a trend towards better OS (p=0.06) with primary ND (22). Other studies did not find a difference in OS, DFS and LRFS between primary surgery and primary non-surgical treatment (23, 28). Conversely, Kamal et al. found excellent regional control rates with intensity modulated RT (IMRT), with no additional benefit of treatment intensification with concomitant CTx or surgery (53). Maghami et al. reported that, with the current body of evidence, decision of treatment modality is most often driven by the nodal status (1). Moreover, trimodal therapy should be maximally avoided. In our tertiary referral center, primary surgery is the preferred initial therapy in the absence of surgical and medical contraindications. Additionally, immune-checkpoint inhibitors (ICI), may be potentially effective as well, as SCCUP patients can be particularly sensitive to ICI by mounting a seemingly more effective anti-tumor immune response (54). The lack of unification in international guidelines emphasizes the need for multicentric randomized controlled trials to attain a consented treatment regimen for SCCUP. Previous literature has criticized CUP research quality, specifically the inadequate sample size for biomarker and validation studies, the lack of preliminary *in-vitro* evidence, and limited high-quality clinical trials. Indeed, there is only a limited number of published clinical trials on this subject, and a significant limitation of current therapeutic studies is that many patients with SCCUP are frequently not included in randomized trials but are treated off study (55). It is widely acknowledged that the quality of evidence regarding SCCUP is rather poor, relying primarily on the variability in the premised definition. We included patients based on a strict definition of SCCUP, although many previous series did not. Therefore comparison of results needs careful interpretation, considering the heterogeneity in patients defined as “SCCUP”.

This study suffers from limitations, including its retrospective nature, low number of included patients, considerable diagnostic and treatment heterogeneity and wide time span of patient inclusion (January 2000 to June 2021). Over time, SCCUP’s diagnostic work up has drastically changed and there has been a shift in etiology. Additionally, the p16 status was not routinely assessed in individuals diagnosed before 2010 which might have led to p16 status turning out a non-significant prognosticator. Moreover, p16 positivity on cytology was not routinely confirmed by HPV DNA *in situ* hybridization (ISH) or PCR: current guidelines of the College of American Pathologists recommend optional confirmatory testing through HPV DNA ISH or PCR when dealing with a cytological sample of a suspected HPV-related cancer as false negative p16 results may be encountered frequently in cytological specimens (56). However, most of our patients underwent primary surgical treatment, allowing a more reliable p16 testing on the surgical specimen. In this case, according to the guidelines of the College of American Pathologists, HPV DNA detection with ISH or PCR is only advised if a SCC is p16-positive without the typical basaloid-like histology or when the primary tumor is found in an anatomic location other than palatine or lingual tonsil (19, 56).

The relatively small cohort size, low number of events (death, recurrence) and considerable amount of patients with unknown p16 status render statistics difficult and demand cautious interpretation of univariable analysis of potential prognosticators in p16-positive and -negative subgroups. Multivariable analysis could therefore only be performed in the overall patient group.

## Conclusion

This retrospective cohort analysis reports oncological outcomes comparable to previous studies. Higher cN status (TNM 7) and presence of cENE/rENE are significant negative predictors for survival and recurrence. Only rENE remained a significant negative prognostic factor in p16 positive and negative subgroup analysis. P16 positivity was not related to better oncological outcomes. These results support the suggested value of rENE as a risk stratification tool in HNSCC.

## Data availability statement

The raw data supporting the conclusions of this article will be made available by the authors, without undue reservation.

## Ethics statement

The studies involving human participants were reviewed and approved by University Hospitals Leuven Committee for Medical Ethics. Written informed consent for participation was not required for this study in accordance with the national legislation and the institutional requirements.

## Author contributions

JM, JV, and VP contributed to study setup, data collection, data quality control, data analysis (statistics), drafting manuscript, and review of manuscript. AL contributed to data quality control, analysis (statistics), drafting manuscript, and review of manuscript. SN, PC, J-FD, CL, and PD contributed to drafting manuscript and review of manuscript. All authors contributed to the article and approved the submitted version.

## Funding

Costs related to statistical analysis and manuscript publication were funded through the Vandeputte Walter Hoofd-Halskanker fund of the KU Leuven.

## Conflict of interest

The authors declare that the research was conducted in the absence of any commercial or financial relationships that could be construed as a potential conflict of interest.

## Publisher’s note

All claims expressed in this article are solely those of the authors and do not necessarily represent those of their affiliated organizations, or those of the publisher, the editors and the reviewers. Any product that may be evaluated in this article, or claim that may be made by its manufacturer, is not guaranteed or endorsed by the publisher.
